# Development of a novel prognostic assessment tool for recurrent respiratory papillomatosis

**DOI:** 10.1186/s12916-026-04832-w

**Published:** 2026-04-16

**Authors:** Satoshi Yamada, Taro Ikegami, Takahiro Fukuhara, Yuto Horichi, Hirotaka Shinomiya, Ken-ichi Nibu, Koji Ebisumoto, Hiroaki Iijima, Akihiro Sakai, Kenji Okami, Koichiro Wasano, Yosuke Nakanishi, Tomokazu Yoshizaki, Takuya Nakagawa, Toyoyuki Hanazawa, Jun Okamura, Yushi Ueki, Arata Horii, Norimoto Kise, Mikio Suzuki, Mariko Kasuga, Shu Yokota, Yutaka Takumi, Hajime Ishinaga, Masako Kitano, Ichiro Ota, Takashi Masui, Nobuhiro Yoshimi, Yutaka Sasaki, Yoshihiro Noda, Sho Morita, Goro Takahashi, Kazutaka Takeuchi, Yuki Nakamura, Kotaro Ishida, Kosuke Sugawara, Shigeru Matsuda, Yusei Makoshi, Natsuki Sugiyama, Kotaro Kano, Sosuke Sahara, Junya Kita, Kotaro Morita, Daiki Mochizuki, Hiroshi Nakanishi, Atsushi Imai, Yuki Misawa, Yoshinori Takizawa, Hideya Kawasaki, Kiyoshi Misawa

**Affiliations:** 1 Department of Otolaryngology/Head and Neck Surgery, Hamamatsu University School of Medicine, 1-20-1, Handayama, Chuo-Ku, Hamamatsu, Shizuoka 431-3192 Japan; 2 Department of Otorhinolaryngology, Head and Neck Surgery, Graduate School of Medicine, University of the Ryukyus, 1076 Kiyuna, Ginowan, Okinawa 901-2725 Japan; 3 Department of Otolaryngology, Head and Neck Surgery, Faculty of Medicine, Tottori University, 36-1, Nishicho, Yonago, 683-8504 Japan; 4 Department of Otolaryngology-Head and Neck Surgery, Jichi Medical University Hospital, Shimotsuke, 329-0498 Japan; 5 Department of Otolaryngology-Head and Neck Surgery, Kobe University School of Medicine, 7-5-1 Kusunoki-Choho, Chuo-Kuu, Kobe, 650-0017 Japan; 6 Department of Otolaryngology, Head and Neck Surgery, Tokai University School of Medicine, 143 Shimokasuya, Isehara, Kanagawa 259-1193 Japan; 7 Division of Otolaryngology, Head and Neck Surgery, Graduate School of Medical Science, Kanazawa University, 13-1 Takara-Machi, Kanazawa, Ishikawa 920-8641 Japan; 8 Department of Otorhinolaryngology, Head and Neck Surgery, Graduate School of Medicine, Chiba University, 1-8-1, Inohana, Chuo-Ku, Chiba, 260-8670 Japan; 9 Department of Otorhinolaryngology, Seirei Hamamatsu General Hospital, 2-12-12, Sumiyoshi, Chuo-Ku, Hamamatsu, Shizuoka 430-8558 Japan; 10 Department of Otolaryngology Head and Neck Surgery, Niigata University Graduate School of Medical and Dental Sciences, 1-757, Asahimachi-Dori, Chuo-Kuu, Niigata City, Niigata, 950-8510 Japan; 11 Department of Otorhinolaryngology-Head and Neck Surgery, Shinshu University School of Medicine, 3-1-1, Asahi, Matsumoto, Nagano 390-8621 Japan; 12 Department of Otorhinolaryngology-Head & Neck Surgery, Mie University Graduate School of Medicine, 2-174 Edobashi, Tsu, Mie 514-8507 Japan; 13 Department of Otolaryngology-Head and Neck Surgery, Kindai University Nara Hospital, 1248-1 Otoda-Cho Ikoma, Nara, 630-0293 Japan; 14 Department of Otolaryngology, Yaizu City Hospital, 1000, Michihara, Yaizu, Shizuoka 425-8508 Japan; 15 Department of Otolaryngology, Numazu City Hospital, 550 Harunoki, Higashishiji, Numazu, , Shizuoka 410-0302 Japan; 16 Department of Otorhinolaryngology, Seirei Mikatahara General Hospital, 3453 Mikatahara-Cho, Chuo-Ku, Hamamatsu, Shizuoka 433-8558 Japan; 17 Department of Otorhinolaryngology, Fujieda Municipal General Hospital, 4-1-11, Surugadai, , Fujieda, Shizuoka 426-8677 Japan; 18 Yamahoshi ENT Clinic, 1-4-6, Shitoro, Chuo-Ku, Hamamatsu, Shizuoka 432-8021 Japan; 19 NanoSuit Research Laboratory, Division of Preeminent Bioimaging Research, Institute of Photonics Medicine, Hamamatsu University School of Medicine, 1-20-1, Handayama, Chuo-Ku, Hamamatsu, Shizuoka 431-3192 Japan

**Keywords:** HARRP Score, D-H Classification, Derkay Score, Recurrent Respiratory Papillomatosis, Human Papillomavirus, NanoSuit-CLEM Method

## Abstract

**Background:**

Recurrent respiratory papillomatosis (RRP), caused by the human papillomavirus (HPV), is associated with an unpredictable clinical course. Although the Derkay Score is widely used to determine clinical severity, its prognostic value has rarely been evaluated. We developed a pathological severity score, the Hamamatsu Recurrent Respiratory Papillomatosis Pathological (HARRP) Score and combined it with the Derkay Score to develop a novel clinicopathological system—the Derkay-HARRP (D-H) Classification. We aimed to validate its prognostic value.

**Methods:**

We retrospectively analyzed 125 patients who were clinically diagnosed with RRP from 16 Japanese institutions, randomly divided into validation (*n* = 38) and test (*n* = 87) cohorts. HPV-typing and immunohistochemistry for HPV-L1, HPV-E4, Ki-67, and p16 were performed. HPV particles were confirmed using the NanoSuit-correlative light and electron microscopy (CLEM) method. Receiver operating characteristic (ROC) curve analysis evaluated marker performance and defined recurrence cut-offs. HARRP and Derkay Scores were further assessed by ROC analysis and Cox proportional hazards models. We stratified patients using the D-H Classification and analyzed disease progression over time.

**Results:**

No significant demographic differences were observed between the two cohorts. Positivity of HPV-L1, HPV-E4, and Ki-67 in the upper third of the epithelium was associated with recurrence. NanoSuit-CLEM confirmed HPV particles in HPV-L1–positive areas, supporting pathological relevance. The HARRP Score was calculated by assigning 1 point each for positivity of HPV-L1, HPV-E4, and ≥ 5% Ki-67-positive cells in the upper third of the epithelium. ROC analysis of the HARRP Score showed areas under the curve (AUCs) of 0.675 (validation) and 0.754 (test), whereas the Derkay Score showed AUCs of 0.709 and 0.834, respectively. The cut-off values were 1 and 4, respectively. Both scores were significant in the Cox analysis (*p* < 0.001). The D-H Classification stratified patients as Severe (both positive scores), Moderate (either positive), or Mild (both negative), with significant differences in relapse-free survival (*p* < 0.001). Severity tended to decrease with repeated surgeries and recurrence was rare in the Mild group. Findings remained consistent in HPV-positive cases only.

**Conclusions:**

Combination of the Derkay and HARRP Scores—the D-H Classification—provides a practical tool for risk stratification and personalized follow-up planning of patients with RRP.

**Supplementary Information:**

The online version contains supplementary material available at 10.1186/s12916-026-04832-w.

## Background

Recurrent respiratory papillomatosis (RRP) often occurs as a result of human papillomavirus (HPV) type 6 or 11 [1]. The clinical course varies widely—some patients with RRP achieve long-term remission after only a few surgical interventions, whereas other patients require lifelong treatment [2]. Although RRP is a benign condition, it carries a significant disease burden. The primary goal of treatment is to achieve remission while preserving quality of life [3], particularly voice function [4]. In addition to surgery, several effective treatment options have been reported, including HPV vaccination [5], cidofovir [6], and bevacizumab [7, 8]. Of these, HPV vaccination is associated with the mildest adverse effects and can effectively reduce disease severity [9]. In its position statement, the American Academy of Otolaryngology–Head and Neck Surgery recommends HPV vaccination for patients with RRP aged 9–45 years [10]. Additionally, cidofovir, an antiviral DNA synthesis inhibitor commonly used to treat conditions such as cytomegalovirus retinitis, inhibits HPV replication. Local cidofovir administration has demonstrated benefits in the reduction of disease severity in patients with RRP [11]. Furthermore, the efficacy of systemic administration of bevacizumab in RRP via reduction of perivascular edema in papilloma lesions has been demonstrated [12]. Its effectiveness has also been reported for lower airway lesions, including those with pulmonary involvement, making it a promising option in patients with severe disease [13, 14]. However, as a molecular targeted therapy, it is associated with adverse effects, and although these adverse effects are generally within acceptable limits, they remain a concern [15].

Although various promising treatment options beyond surgery have been reported, an accurate assessment of disease severity is essential for making appropriate treatment choices. The Derkay Score is a widely used clinical tool for evaluating disease severity based on endoscopic findings [16]. This tool is simple to apply in general otolaryngology clinics and it allows repeated assessments. Inter-rater variability falls within statistically acceptable limits; however, the lack of explicit criteria for elements, such as tumor size, renders this assessment subjective [17].

Recent studies have explored RRP disease severity from an etiological perspective. RRP is commonly associated with HPV types 6 and 11, and the detection of HPV DNA in the laryngeal mucosa during remission suggests that persistent HPV infection plays a key role in the disease pathogenesis [18]. Advanced scanning electron microscopy techniques, such as NanoSuit-correlative light and electron microscopy (CLEM) [19–21], have revealed the presence of HPV particles filling HPV-L1-positive nuclei [22, 23], strongly supporting the role of persistent HPV infection in RRP. Furthermore, immunohistochemical (IHC) detection of HPV-L1 is associated with a higher frequency of surgical interventions and shorter intervals between surgeries, marking patients with HPV-L1 as more severe cases [22]. Additionally, increased mRNA expression of E4, which is involved in HPV particle formation, has been reported in laryngeal papillomatosis [24, 25]. Other factors influencing disease severity include HPV type 11 [26, 27] involvement and juvenile onset [28, 29]. Furthermore, HPV-negative papillomas are generally not classified as RRP because their etiology and clinical severity differ from those of typical HPV-positive RRP [30]. Against this background, HPV testing has recently been recommended in the United States for diagnostic and clinical evaluation of RRP; however, HPV testing is not yet widely adopted outside the United States [31].

In this multi-institutional study involving patients from 16 facilities in Japan, we evaluated several IHC markers related to RRP etiology, including HPV-L1 for viral particle formation, HPV-E4 for high mRNA expression, MIB-1 for tumor proliferation, and p16, a surrogate marker for HPV-related oropharyngeal cancer [32]. Significant IHC findings were used to develop a novel pathological scoring system named the Hamamatsu Recurrent Respiratory Papillomatosis Pathological (HARRP) Score. Furthermore, by integrating the pathologically assessed HARRP Score with the clinically assessed Derkay Score, we developed a novel severity stratification system for RRP named the Derkay-HARRP (D-H) Classification. The HARRP Score and the D–H classification were developed using the entire cohort of papillomatous lesions without distinguishing between HPV-positive and HPV-negative cases, reflecting real-world clinical practice. In addition, analyses were performed specifically in an HPV-positive population with RRP in mind. We aimed to assess the utility of the HARRP Score and D-H Classification for prediction of disease prognosis.

## Methods

### Study participants and tumor samples

From January 1, 2009, to December 31, 2023, we analyzed 139 patients who were clinically diagnosed with RRP underwent complete tumor resection, with subsequent pathological diagnosis of papilloma. The aim of all surgeries was to achieve remission and cases involving debulking procedures were excluded. However, the surgical approach was not restricted. Patients were enrolled across 16 facilities across Japan, including the lead facility, Hamamatsu University School of Medicine. The study was conducted in accordance with the principles of the Declaration of Helsinki. Approval was obtained from the Institutional Review Board (IRB) of Hamamatsu University School of Medicine (approval number: 19–222). Ethical approval was secured individually from each participating institutions. Facility names and approval numbers are summarized in Additional file 1: Table S1.

**Fig. 1:**
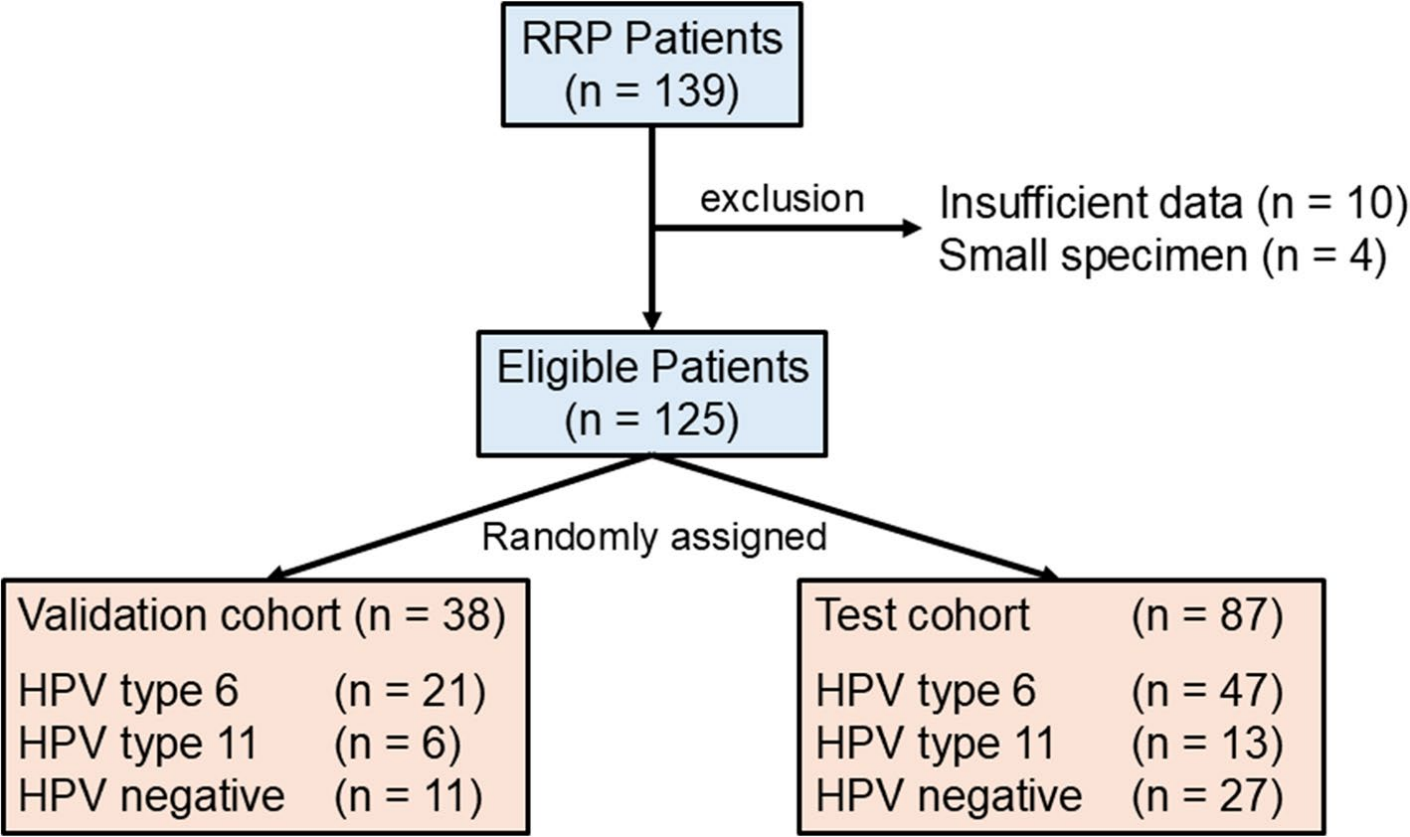
Enrollment and allocation of study participants. In total, 139 patients are enrolled in this study. After excluding 10 patients with insufficient data and four patients with small sample sizes, 125 patients are included in the final analysis. These patients are randomly assigned to either the validation cohort (*n* = 38) or test cohort (*n* = 87)

To evaluate the HARRP Score and D-H Classification, we used surgical specimens from the initial procedures performed during the study period. Four patients with small specimens that were insufficient for accurate pathological assessment were excluded. Additionally, 10 patients with incomplete clinical information were excluded from the analysis. To develop the HARRP Score, IHC results were used to establish parameters in a validation cohort (n = 38) and reproducibility was confirmed using a test cohort (n = 87) (Fig. 1). Stratified randomization was performed using R software (ver. 4.4.2; R Foundation for Statistical Computing, Vienna, Austria) and the splitTools package was used. Furthermore, to analyze temporal changes in the D-H Classification, we examined all patients during the study period.

The clinical data collected included age, sex, smoking history, alcohol consumption, Derkay Score, number of surgeries, surgical dates, tumor recurrence dates, final follow-up dates, and the presence or absence of malignant transformation.

### HPV genotyping

DNA was extracted from fresh papilloma tissues obtained during surgery using the QIAamp DNA Mini Kit (Qiagen, Hilden, Germany). In cases where only formalin-fixed paraffin-embedded (FFPE) tissues were available, DNA was extracted using the QIAamp DNA FFPE Tissue Kit (Qiagen, Hilden, Germany). HPV was detected by polymerase chain reaction (PCR) using the TaKaRa PCR Human Papillomavirus Typing Set (Takara Bio, Kusatsu, Japan).

For HPV-positive patients, direct sequencing analysis was conducted using the 3500xL Genetic Analyzer (Applied Biosystems, Waltham, MA, USA). The sequences obtained were aligned using BLAST (https://blast.ncbi.nlm.nih.gov/) to determine HPV genotypes.

#### IHC

FFPE tissues were sectioned into 4-μm thick slices. Epitope Retrieval Solution (pH 9; Leica Biosystems, Wetzlar, Germany) was used for antigen retrieval. The incubation conditions were as follows: 95 °C for 30 min for HPV-L1, HPV type 6 E4, HPV type 11 E4, and p16; and 121 °C for 20 min using an autoclave for Ki-67. To block endogenous peroxidase activity, sections were incubated in a methanol solution containing 0.3% H_2_O_2_ for 10 min. The following primary antibodies were used: HPV-L1 (K1H8) (Thermo Fisher Scientific, Waltham, MA, USA), HPV type 6 E4 [25], HPV type 11 E4 [25], p16 (E4H6) (Roche, Basel, Switzerland), and Ki-67 (MIB-1) (DAKO, Santa Clara, CA, USA). HPV-L1 was incubated at 4 °C overnight, whereas the other antibodies were incubated at room temperature for 30 min. For secondary antibodies, MAX-PO(M) (Nichirei Bioscience, Tokyo, Japan) was used for HPV-L1, HPV type 6 E4, p16, and Ki-67, whereas MAX-PO(R) (Nichirei Bioscience, Tokyo, Japan) was used for HPV type 11 E4. All the secondary antibody reactions were performed at room temperature for 30 min. Color development was achieved using a 3,3'-diaminobenzidine (DAB) kit (Nichirei Bioscience, Tokyo, Japan), and counterstaining was performed using Mayer’s hematoxylin (Nichirei Bioscience, Tokyo, Japan).

### HPV particle observation using NanoSuit-CLEM

HPV-L1 IHC was performed and the slides were digitized using a NanoZoomer 2.0-HT virtual slide scanner (Hamamatsu Photonics, Shizuoka, Japan). The positions for field emission scanning electron microscopy (FE-SEM) observations were confirmed using NDP.view2 software (Hamamatsu Photonics, Shizuoka, Japan) as a reference. Subsequently, the slide glass with HPV-L1 IHC was incubated with xylene to remove the cover glass. The slides were sequentially incubated in 100%, 95%, 80%, and 70% ethanol, followed by incubation in distilled water for hydration treatment. The slides were incubated in 1% osmium tetroxide for 5 min. Osmium binds specifically to DAB, allowing enhanced images to be obtained during FE-SEM observation. NanoSuit Type II solution (200 μl; Nisshin EM, Tokyo, Japan) was dropped onto the slide. The solution was spread thinly on a glass slide by centrifugation at 3000 rpm for 15 s using a spin-coater.

FE-SEM was performed using a Hitachi S-4800 field-emission scanning electron microscope (Hitachi, Tokyo, Japan). The accelerating voltage was set to 5.0 kV. Images were obtained in the yttrium–aluminum-garnet backscattered electron (YAG-BSE) mode.

### Pathological analysis

IHC- and hematoxylin and eosin (HE)-stained specimens obtained from postoperative pathological examinations were digitized using a NanoZoomer 2.0-HT virtual slide scanner (Hamamatsu Photonics, Shizuoka, Japan). Images were analyzed using NDP.view2 software (Hamamatsu Photonics, Shizuoka, Japan). The IHC evaluation was performed by pathologists who were blinded to clinical information.

IHC for HPV-L1, HPV type 6 E4, HPV type 11 E4, and p16 was performed at 20 × magnification using NDP.view2 software (Hamamatsu Photonics, Shizuoka, Japan). Positive regions were counted and scored as follows: negative (0 points), positive in one region (1 point), positive in two regions (2 points), or positive in three or more regions (3 points).

Ki-67 expression was assessed using the MIB-1 index, which measures the proportion of tumor cells with positive nuclear staining. The area with the highest positivity rate was identified and recorded using NDP.view2 software at 10 × magnification. The MIB-1 index was calculated using the “Positive Cell Detection” function in QuPath (https://qupath.github.io/). The parameters used for the analysis are summarized in Additional file 2: Table S2. For the MIB-1 index, the tumor was divided into three layers based on its depth from the basal layer. Only the upper third, which corresponded to the most superficial layer, was measured. Additionally, measurements were performed for the entire tumor.

### Statistical analysis

A patient background analysis was conducted by comparing the validation and test cohorts. Age, Derkay Score, and number of surgeries performed prior to study enrollment were evaluated using the Mann–Whitney U test. A Fisher’s exact test was performed for categorical variables, such as sex, smoking, alcohol consumption, and malignant transformation. IHC, HARRP Score, and Derkay Score results were analyzed using receiver operating characteristic (ROC) curve analysis to evaluate their ability to predict recurrence, and the area under the curve (AUC) was calculated. Cut-off values were determined based on the Youden index. To assess the prognostic value of recurrence, Cox proportional hazards models were applied, adjusting for age (≥ 60 years vs. < 60 years), sex, alcohol consumption, smoking, Derkay Score (≥ 4 vs. < 4), and HARRP Score (≥ 1 vs. < 1). Additionally, relapse-free survival was evaluated using Kaplan–Meier curves for the HARRP Score, Derkay Score, and D-H Classification. Statistical significance was assessed using log-rank tests. To evaluate the scores under conditions reflecting real-world clinical practice where routine HPV testing is challenging, ROC curve analysis, Cox proportional hazards models, and Kaplan–Meier curves were first performed including HPV-negative cases. Subsequently, because RRP is generally HPV-positive, analyses were repeated focusing only on HPV-positive cases. Statistical analyses were performed using SPSS version 29.0.2.0(20) (IBM Corp., Armonk, NY, USA) and GraphPad Prism version 10.5.0 (774) (GraphPad Software, Boston, MA, USA). Statistical significance was set at *p* < 0.05.

## Results

### Patient characteristics

Patient characteristics are summarized (Table 1). No statistically significant differences were observed between the validation and test cohorts for any background characteristics. The HPV genotypes detected in patients were exclusively HPV types 6 or 11, with no other types identified and no patients were co-infected with both types. Two cases were identified in which PCR was negative, but both HPV type 6 E4 and HPV L1 were positive by IHC. These cases were classified as HPV type 6 positive. No cases were observed in which PCR was negative, and only one of HPV E4 or HPV L1 was positive by IHC.

**Table 1:** Patient characteristics

			Validation cohort (*n* = 38)	Test cohort (*n* = 87)	*p*
Age mean (range, SD)	all		51.10 (9–86, 16.24)	53.23 (4–88, 17.36)	0.451
	HPV type 6		43.90 (9–71, 14.90)	49.06 (9–88, 16.37)	0.322
	HPV type 11		51.83 (37–66, 10.42)	48.69 (4–75, 19.10)	0.765
	HPV-negative		64.45 (42–86, 13.12)	62.59 (32–81, 14.58)	0.987
Sex	all	M	33	74	
		F	5	13	> 0.999
	HPV type 6	M	19	38	
		F	2	9	0.482
	HPV type 11	M	5	12	
		F	1	1	0.544
	HPV-negative	M	9	24	
		F	2	3	0.615
Smoking	all	+	26	60	
		-	12	27	> 0.999
	HPV type 6	+	14	29	
		-	7	18	0.789
	HPV type 11	+	3	11	
		-	3	2	0.262
	HPV-negative	+	9	20	
		-	2	7	0.700
Alcohol	all	+	26	66	
		-	12	21	0.387
	HPV type 6	+	16	34	
		-	5	13	> 0.999
	HPV type 11	+	4	11	
		-	2	2	0.561
	HPV-negative	+	6	21	
		-	5	6	0.229
Derkay Score mean (range, SD)	all		4.82 (1–16, 3.32)	4.91 (1–13, 2.71)	0.489
	HPV type 6		5.67 (2–16, 3.58)	5.85 (2–13, 2.80)	0.517
	HPV type 11		4.67 (2–12, 1.50)	4.54 (3–6, 1.20)	0.282
	HPV-negative		3.27 (1–8, 2.05)	3.44 (1–10, 2.44)	0.910
No. of surgeries before the study mean (range, SD)	all		0.52 (0–5, 1.22)	1.03 (0–21, 2.81)	0.36
	HPV type 6		0.67 (0–4, 1.24)	1.55 (0–21, 3.44)	0.29
	HPV type 11		1.00 (0–5, 2.00)	0.31 (0–2, 0.75)	0.579
	HPV-negative		0 (0, 0)	0.48 (0–10, 1.93)	0.194
Malignant transformation	all	+	0	3	
		-	38	84	0.551
	HPV type 6	+	0	0	
		-	21	47	-
	HPV type 11	+	0	0	
		-	6	13	-
	HPV-negative	+	0	3	
		-	11	24	0.542

Age, Derkay Score, and number of surgeries performed prior to study enrollment are evaluated using the Mann–Whitney U test. A Fisher’s exact test is performed for categorical variables, such as sex, smoking, alcohol consumption, and malignant transformation. *HPV* Human papillomavirus, *SD* Standard deviation

The mean age of the patients in the validation cohort was 51.10 years (range: 9–86 years, SD: 16.24), whereas that in the test cohort was 53.23 years (range: 4–88 years, SD: 17.36). Among patients who were HPV-negative, the mean age was 64.45 years (range: 42–86, SD: 13.12) in the validation cohort and 62.59 years (range: 32–81, SD: 14.58) in the test cohort. Given that the mean age of patients with HPV types 6 or 11 was late 40 s to 50 s, patients who were HPV-negative were predominantly older (*p* < 0.001). Notably, none of the patients who were HPV-negative had juvenile-onset RRP. Regardless of HPV status, male patients were more common, and a high proportion had a history of alcohol consumption and smoking. Notably, patients with detectable HPV exhibited higher Derkay Scores (*p* < 0.001) and pre-study surgical counts (*p* = 0.004), indicating greater clinical severity. However, all patients with malignant transformation were HPV-negative. These findings are consistent with those of previous reports and reinforce the known associations with patient background.

### Pathological findings

**Fig. 2:**
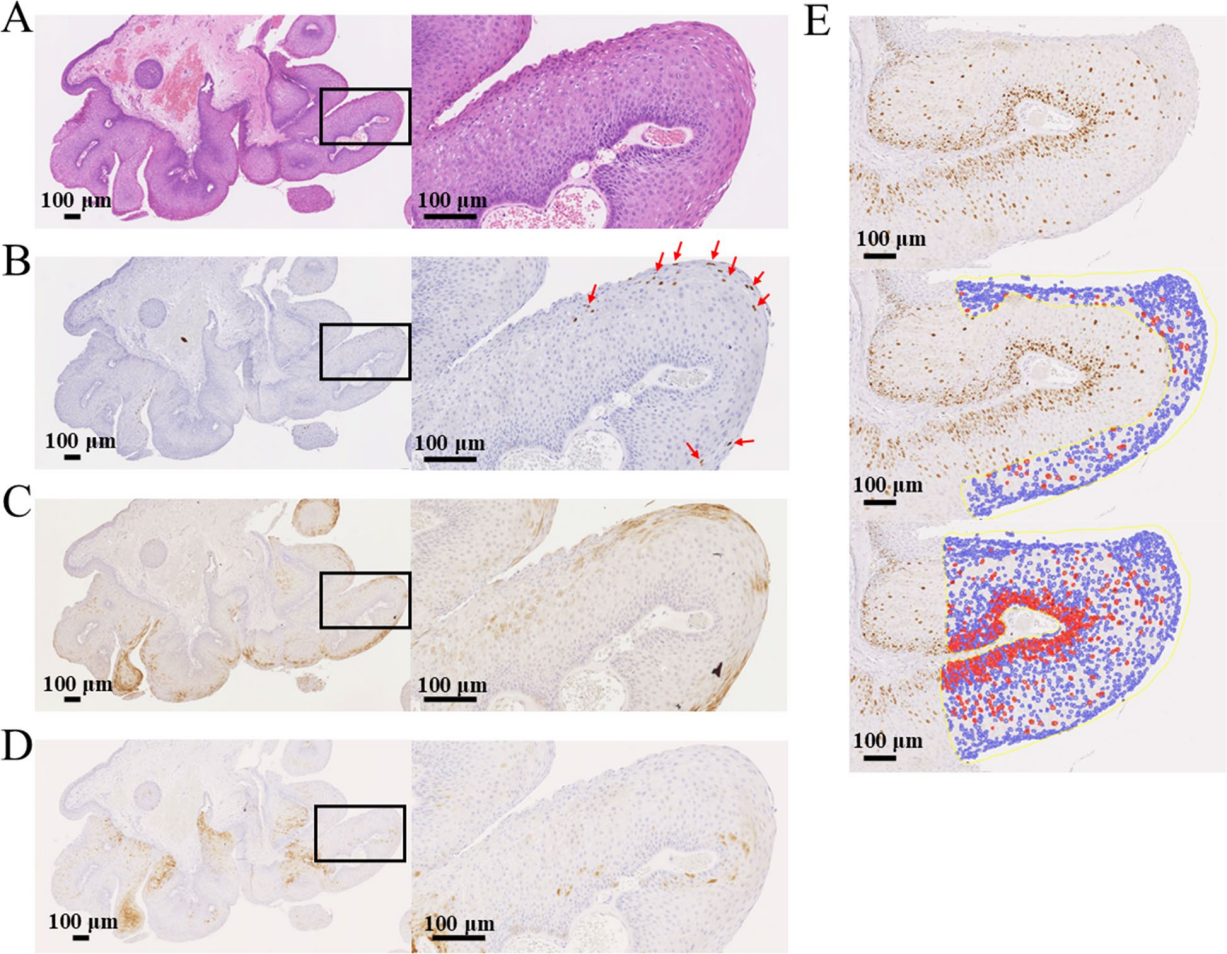
Pathological findings and assessment methods. **A** HE staining. The tumor exhibits papillary proliferation from the basal layer. **B** IHC for HPV-L1. Positive nuclear staining in the superficial layer of the tumor. **C** IHC for HPV-E4. Positive cytoplasmic staining in the superficial layer of the tumor, showing a distribution similar to that of HPV-L1. **D** IHC for p16. Diffuse, strong positivity is not observed; instead, scattered positive cells are observed. **E** IHC for Ki-67 and evaluation of Ki-67 positivity is observed in nearly all basal layer cells, with a gradual decrease in positive cells toward the superficial layers (upper panel). The MIB-1 index is measured in both the upper third of the tumor (middle panel) and the entire tumor (lower panel). Blue indicates Ki-67-negative cells, and red indicates Ki-67-positive cells. For images A–D, the left panels show low-magnification views (× 5), and the boxed areas are shown at a higher magnification (× 20) in the right panels. Scale bars in each image represent 100 μm. HE, hematoxylin and eosin; IHC, immunohistochemistry; HPV, human papillomavirus

HE staining revealed that the tumors proliferated in a papillary pattern originating from the basal layer. Typically, the superficial layers of the tumors exhibited koilocytosis, characterized by cytoplasmic ballooning and nuclear atypia (Fig. 2A).

The superficial tumor layers were positive for the HPV capsid protein, HPV-L1, predominantly in areas of koilocytosis (Fig. 2B). Similarly, HPV-E4, which is associated with HPV particle production, showed a staining pattern similar to that of HPV-L1 (Fig. 2C). Among the 125 patients, HPV-L1 was positive in 24 patients (20.0%) and HPV-E4 was positive in 49 patients (39.2%). Restricting the analysis to patients who were HPV-positive, the positivity rates were 27.5% for HPV-L1 and 56.3% for HPV-E4. In patients who were HPV-negative, both HPV-L1 and HPV-E4 were negative, indicating that approximately one-fourth of the patients who were HPV-positive were HPV-L1 positive, whereas approximately half were HPV-E4 positive.

IHC for p16, which is widely used as a surrogate marker for HPV infection in HPV-associated oropharyngeal cancers [32], showed faint, scattered positivity predominantly in tumor tissues in patients with RRP, regardless of HPV status (Fig. 2D).

Ki-67 IHC showed the highest expression in the basal layers of the tumors, with a decreasing positivity gradient toward the superficial layers. This pattern was consistent across both patients who were HPV-positive and those show were HPV-negative. The MIB-1 index for the upper third of the tumor surface and the entire tumor was measured using QuPath software. Blue and red dots represent negative and positive cells, respectively (Fig. 2E).

### HPV particles in HPV-L1 IHC-positive areas

**Fig. 3:**
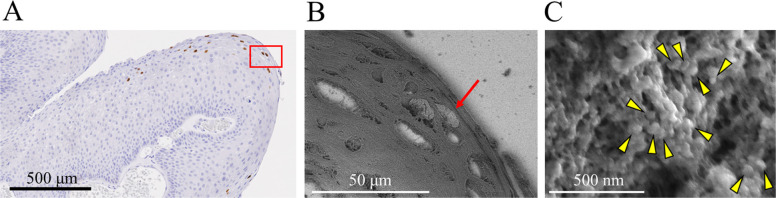
HPV particle observation using the NanoSuit-CLEM method. **A** IHC for HPV-L1. The area for observation is selected using the NanoSuit-CLEM method; in this case, the region marked by the red square is chosen. **B** SEM observations using the NanoSuit-CLEM method. The same region is identified as in (**A**). HPV-L1-positive cells appear as bright-enhanced structures. **C** The area indicated by the arrow in (**B**) observed at 100,000 × magnification. Numerous fine particles measuring approximately 50–60 nm, corresponding to the size of HPV virions, are observed filling the area. Scale bars represent: A, 500 μm; B, 50 μm; C, 500 nm. IHC, immunohistochemistry; HPV, human papillomavirus; CLEM, correlative light and electron microscopy; SEM, scanning electron microscopy

HPV-L1 IHC was performed and positive areas were confirmed (Fig. 3A) using the NanoSuit-CLEM method (Fig. 3B). Nuclei showing HPV-L1 positivity reacted with osmium tetroxide, resulting in enhanced white images. When the nucleus of the cell was observed at 100,000 × magnification, numerous particles measuring 50–60 nm were detected (Fig. 3C). These particles were a similar size to that of HPV particles, in agreement with our previous reports. Based on these findings, HPV particles were considered to exist in regions showing HPV-L1 IHC positivity.

### Development of the HARRP Score

**Fig. 4:**
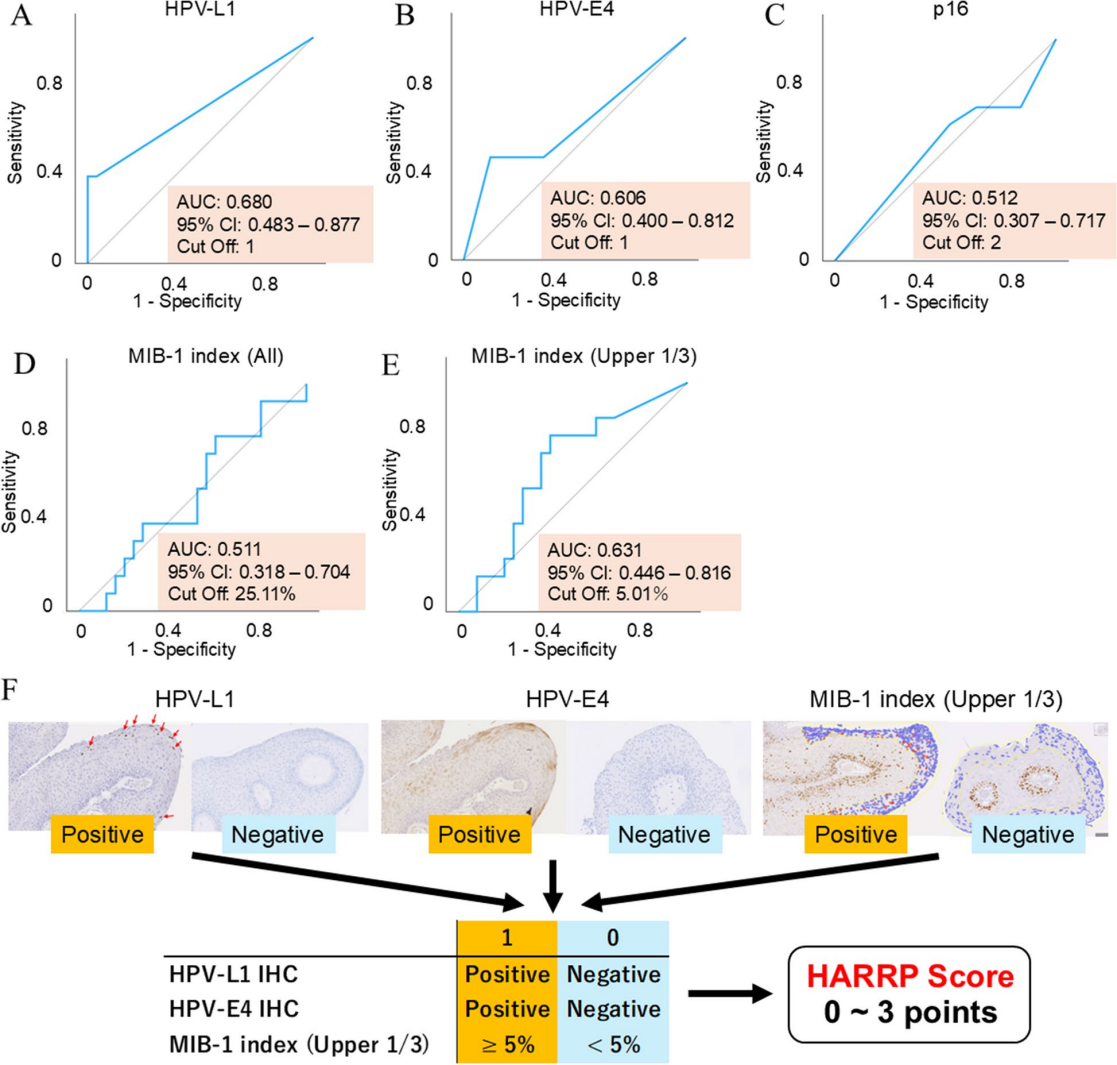
Predication of recurrence using IHC and definition of the HARRP Score. Analyses are performed in the validation cohort (n = 38). **A** The AUC for HPV-L1 is 0.680 (95% CI: 0.483–0.837), with a cut-off value of 1. **B** The AUC for HPV-E4 is 0.606 (95% CI: 0.400–0.812) with a cut-off value of 1. **C** The AUC for p16 is 0.512 (95% CI: 0.307–0.717), with a cut-off value of 2. **D** The AUC for the MIB-1 index is 0.511 (95% CI: 0.316–0.704) with a cut-off value of 25.11%. **E** The AUC for the MIB-1 index in the upper one-third of the tumor is 0.631 (95% CI: 0.446–0.816) with a cut-off value of 5.01%. **F** The HARRP Score is composed of HPV-L1, HPV-E4, and MIB-1 indices in the upper third of the tumor. If HPV-L1 and HPV-E4 are positive, they are each allocated 1 point, and if the MIB-1 index in the upper one-third of the tumor is positive at 5% or more, it is allocated 1 point. The HARRP Score is evaluated based on the total points and scores range from 0 to 3. IHC, immunohistochemistry; HPV, human papillomavirus; HARRP Score, Hamamatsu Recurrent Respiratory Papillomatosis Pathological Score; AUC, area under the curve; CI, confidence interval

We used surgical specimens from the baseline assessments of 38 patients in the validation cohort. ROC curves were generated to predict recurrence based on IHC scores and positivity rates (Fig. 4A-E). HPV-L1 (AUC: 0.680, 95% confidence interval [CI]: 0.483–0.877, cut-off: 1), HPV-E4 (AUC: 0.606, 95% CI: 0.400–0.812, cut-off: 1), and the superficial one-third of the MIB-1 index (AUC: 0.631, 95% CI: 0.446–0.816, cut-off: 5.01%) showed low predictive accuracy for recurrence when used individually. The cut-off values for HPV-L1 and HPV-E4 were both 1, indicating that even the slightest positive result would reach the cut-off value. In contrast, p16 (AUC: 0.512, 95% CI: 0.307–0.717, cut-off: 2) and the overall tumor MIB-1 index (AUC: 0.511; 95% CI: 0.318–0.704, cut-off: 25.11%) were ineffective for predicting recurrence.

We further evaluated whether similar results could be obtained when the analysis was restricted to the 27 HPV-positive cases in the validation cohort (21 HPV type 6–positive and 6 HPV type 11–positive cases) (Additional file 3: Fig. S1A-E). The predictive accuracy for recurrence based on ROC analysis was as follows: HPV-L1 (AUC: 0.710, 95% CI: 0.495–0.926, cut-off: 1), HPV-E4 (AUC: 0.594, 95% CI: 0.356–0.832, cut-off: 1), p16 (AUC: 0.503, 95% CI: 0.272–0.734, cut-off: 2), MIB-1 index (AUC: 0.534, 95% CI: 0.314–0.755, cut-off: 31.78%), and the superficial one-third of the MIB-1 index (AUC: 0.693, 95% CI: 0.487–0.899, cut-off: 5.30%). Overall, the findings were largely comparable to those obtained when HPV-negative cases were included.

These results led to the inclusion of HPV-L1, HPV-E4, and the superficial one-third of the MIB-1 index in the HARRP Score, each of which was associated with recurrence. Each component was assigned 1 point if it met or exceeded the cut-off value. Specifically, HPV-L1 and HPV-E4 were assigned 1 point each if they showed positive results on IHC, and the superficial one-third of the MIB-1 index was assigned 1 point if it showed a positive result of 5% or more. The total HARRP Scores ranged from 0 to 3 points (Fig. 4F).

### Prediction of RRP recurrence using the Derkay and HARRP Scores

**Fig. 5:**
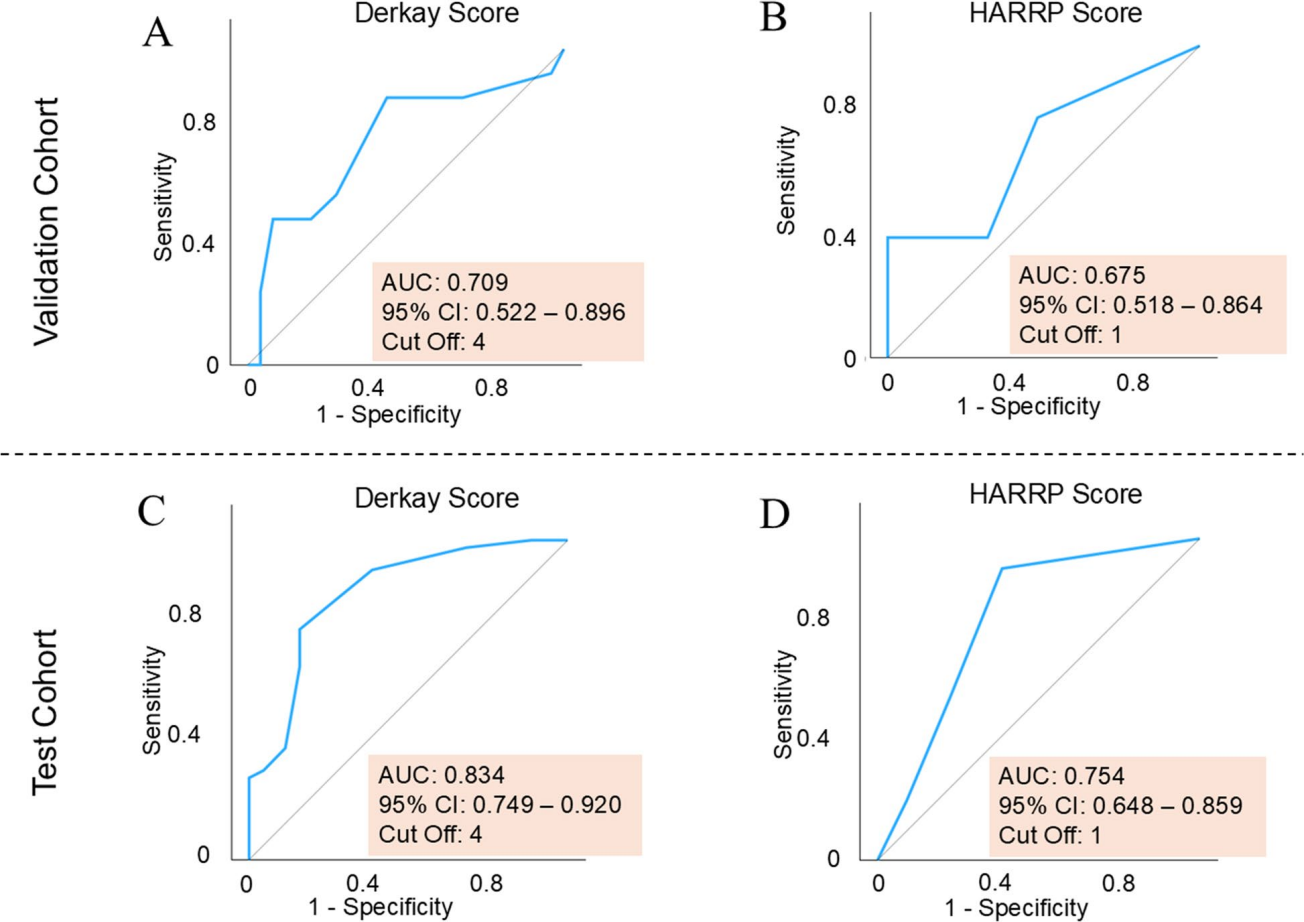
Prediction of recurrence and reproducibility of the Derkay and HARRP Scores. **A** In the validation cohort, the AUC of the Derkay Score is 0.709 (95% CI: 0.522–0.896) with a cut-off value of 4. **B** In the validation cohort, the AUC of the HARRP Score is 0.675 (95% CI: 0.518–0.864) with a cut-off value of 1. **C** In the test cohort, the AUC of the Derkay Score is 0.834 (95% CI: 0.749–0.920) with a cut-off value of 4. **D** In the test cohort, the AUC of the HARRP Score is 0.754 (95% CI, 0.648–0.859) with a cut-off value of 1. These findings suggest that both the Derkay and HARRP Scores are reproducible and serve as useful predictors of recurrence. HARRP Score, Hamamatsu Recurrent Respiratory Papillomatosis Pathological Score; AUC, area under the curve

Subsequently, we evaluated the predictive accuracy of the Derkay and HARRP Scores for RRP recurrence using ROC curve analysis (Fig. 5A–D). In the test cohort, the Derkay Score demonstrated an AUC of 0.709 (95% CI: 0.552–0.896, cut-off: 4), whereas the HARRP Score yielded an AUC of 0.675 (95% CI: 0.518–0.864, cut-off: 1). In the validation cohort, the Derkay Score showed an AUC of 0.834 (95% CI: 0.749–0.920, cut-off: 4) with a cut-off value of 1, and the HARRP Score had an AUC of 0.754 (95% CI: 0.648–0.859, cut-off: 1) with the same cut-off value. These results indicate that both scoring systems are capable of predicting recurrence and that their performance is largely reproducible across independent cohorts.

We evaluated the predictive performance of the Derkay and the HARRP scores for recurrence, restricting the analysis to HPV-positive cases in both the validation and test cohorts (Additional file 4: Fig. S2A-D). In the validation cohort, the AUC values were 0.787 (95% CI: 0.602–0.972; cut-off: 4) for the Derkay score and 0.713 (95% CI: 0.508–0.918; cut-off: 1) for the HARRP score. In the test cohort, the corresponding values were 0.792 (95% CI: 0.669–0.916; cut-off: 4) for the Derkay score and 0.709 (95% CI: 0.569–0.849; cut-off: 1) for the HARRP score. These results suggest that the predictive performance of both scores is reproducible even when restricted to HPV-positive cases. Furthermore, the optimal cut-off values remained unchanged regardless of HPV infection status (cut-off: 4 for the Derkay score and 1 for the HARRP score), and the AUC values were comparable to those obtained in analyses including all cases.

Furthermore, we examined the contribution of each score to the recurrence risk using a Cox proportional hazards model (Table 2). In univariate analysis, variables such as age (≥ 60 years vs. < 60 years), sex, alcohol consumption, and smoking status were not significantly associated with recurrence. In contrast, the Derkay Score (≥ 4 vs. < 4) demonstrated a hazard ratio (HR) of 12.202 (95% CI: 4.609–32.306, *p* < 0.001), and the HARRP Score (≥ 1 vs. < 1) also showed an HR of 12.202 (95% CI: 3.828–24.350, *p* < 0.001). In multivariate stepwise analysis, the Derkay Score remained a significant predictor with an HR of 8.455 (95% CI: 3.026–23.624, *p* < 0.001), while the HARRP Score showed an HR of 6.416 (95% CI: 2.366–17.399, *p* < 0.001). We also evaluated recurrence risk using a Cox proportional hazards model restricted to HPV-positive cases(Additional file 5: Table S3). In the univariate analysis, the Derkay score was associated with a hazard ratio (HR) of 4.294 (95% CI: 1.533–12.03, *p* < 0.006), and the HARRP score had an HR of 3.528 (95% CI: 1.262–9.864, *p* < 0.016). In the multivariate stepwise analysis, the Derkay score remained significantly associated with recurrence (HR 4.109, 95% CI: 1.466–11.520, *p* < 0.007), as did the HARRP score (HR 3.339, 95% CI: 1.194–9.339, *p* < 0.022).

**Table 2:** Cox proportional hazards analysis of Derkay and HARRP Scores in relation to recurrence

Covariate	Univariate analysis		Multivariate stepwise analysis	
	HR (95% CI)	p	HR (95% CI)	p
Age	0.616 (0.295–1.285)	0.196	-	-
Sex	1.328 (0.478–3.686)	0.587	-	-
Alcohol	1.604 (0.707–3.638)	0.258	-	-
Smoking	0.680 (0.321–1.441)	0.314	-	-
Derkay Score	12.202 (4.609–32.306)	< 0.001***	8.455 (3.026–23.624)	< 0.001***
HARRP Score	9.655 (3.828–24.350)	< 0.001***	6.416 (2.366–17.399)	< 0.001***

Cox proportional hazards models are applied, adjusting for age (≥ 60 years vs. < 60 years), sex, alcohol consumption, smoking, Derkay Score (≥ 4 vs. < 4), and HARRP Score (≥ 1 vs. < 1). ***: *p* < 0.001. *HARRP Score* Hamamatsu Recurrent Respiratory Papillomatosis Pathological Score, *CI* Confidence interval, *HR* Hazard ratio

We further examined the correlation between the Derkay score and the HARRP score (Additional file 6: Fig. S3A-H). The correlation coefficient between the two scores was *r* = 0.388, and when restricted to HPV-positive cases, it was *r* = 0.105. These results indicate a weak positive correlation between the Derkay Score and the HARRP Score. In the Cox proportional hazards model, the Derkay Score and the HARRP Score were identified as independent risk factors for recurrence. This supports the interpretation that the Derkay Score, based on clinical information, and the HARRP Score, derived from pathological findings, reflect different aspects of disease status. We also examined the correlation between the Derkay score and each parameter included in the HARRP score. The correlation coefficients were *r* = 0.192 for L1-IHC, *r* = 0.221 for E4-IHC, and *r* = 0.307 for the superficial one-third of the MIB-1 index. When restricted to HPV-positive cases, the correlation coefficients were *r* = 0.116 for L1-IHC, *r* = 0.076 for E4-IHC, and *r* = 0.128 for the superficial one-third of the MIB-1 index.

Although the Derkay Score demonstrated slightly superior predictive performance, both scores were identified as significant contributors to recurrence risk. These findings confirmed the clinical utility of the Derkay Score, which is based on clinical information, and the HARRP Score, which is derived from pathological evaluations, regardless of HPV status.

### Stratification of disease severity

**Fig. 6:**
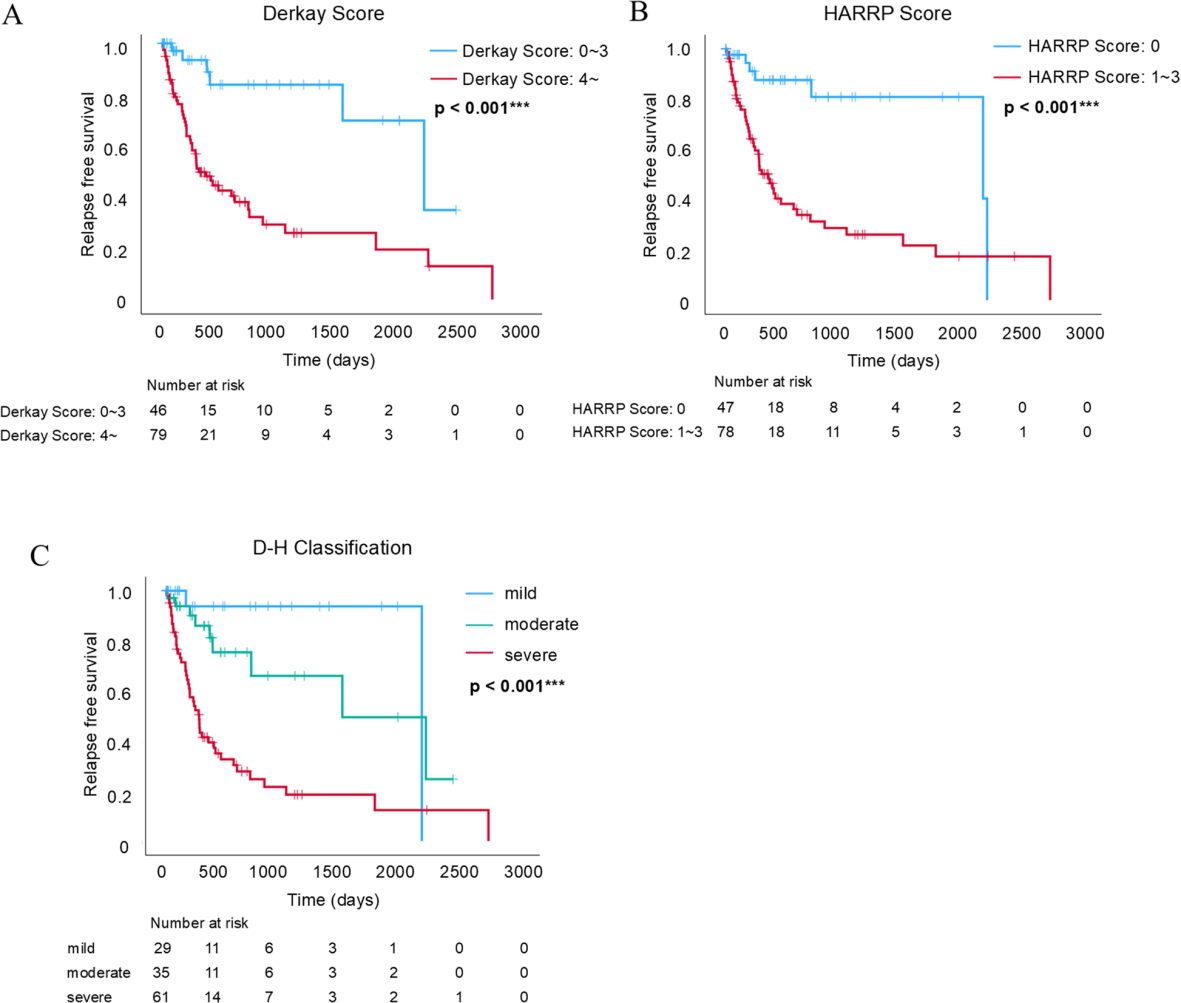
Evaluation of relapse-free survival, **A** Kaplan–Meier analysis based on the Derkay Score. Patients are stratified into two groups: those with scores below the cut-off (0–3 points) and those with scores of 4 or higher. A significant difference in relapse-free survival is observed between the two groups (p < 0.001). **B** Kaplan–Meier analysis based on the HARRP Score. Patients are divided into two groups: those with a score of 0 and those with scores of 1–3. A significant difference in relapse-free survival is observed (p < 0.001). **C** Kaplan–Meier analysis based on the D-H Classification. Patients are categorized into three groups: Mild, Moderate, and Severe. A significant difference in relapse-free survival is found among the three groups (p < 0.001). HARRP Score, Hamamatsu Recurrent Respiratory Papillomatosis Pathological Score; D-H Classification, Hamamatsu Recurrent Respiratory Papillomatosis Pathological (HARRP) Score combined with the Derkay Score Classification

Relapse-free survival was assessed using Kaplan–Meier curves. Patients were stratified into two groups based on whether their Derkay and HARRP Scores were above or below the respective cut-off values derived from the ROC curve analysis. As expected, both scoring systems clearly distinguished between the two groups, with statistically significant differences observed on log-rank tests (*p* < 0.001 for both; Fig. 6A and B). Even when the analysis was restricted to HPV-positive cases, similar results were obtained for the Derkay score and the HARRP score (log-rank tests: Derkay score, *p* < 0.003; HARRP score, *p* < 0.010) (Additional file 7: Fig. S4A and B).

Furthermore, we developed and evaluated the D-H Classification, a novel system combining the Derkay and HARRP Scores. The patients were stratified into three groups: Severe, in which both scores exceeded the respective cut-off values; Moderate, in which only one score exceeded the cut-off value; and Mild, in which both scores were below the cut-off value. Kaplan–Meier analysis of relapse-free survival revealed that this three-tier classification effectively stratified patients according to disease severity (log-rank tests: *p* < 0.001) (Fig. 6C). Similarly, in the D–H classification, restriction to HPV-positive cases still allowed clear stratification of recurrence curves into three groups: mild, moderate, and severe, with statistically significant differences among the groups (log-rank tests: *p* < 0.001) (Additional file 7: Fig. S4C). These findings suggest that integrating clinical and pathological information enables more precise stratification of disease prognosis.

### Clinical course stratified by disease severity using the D-H classification

**Fig. 7:**
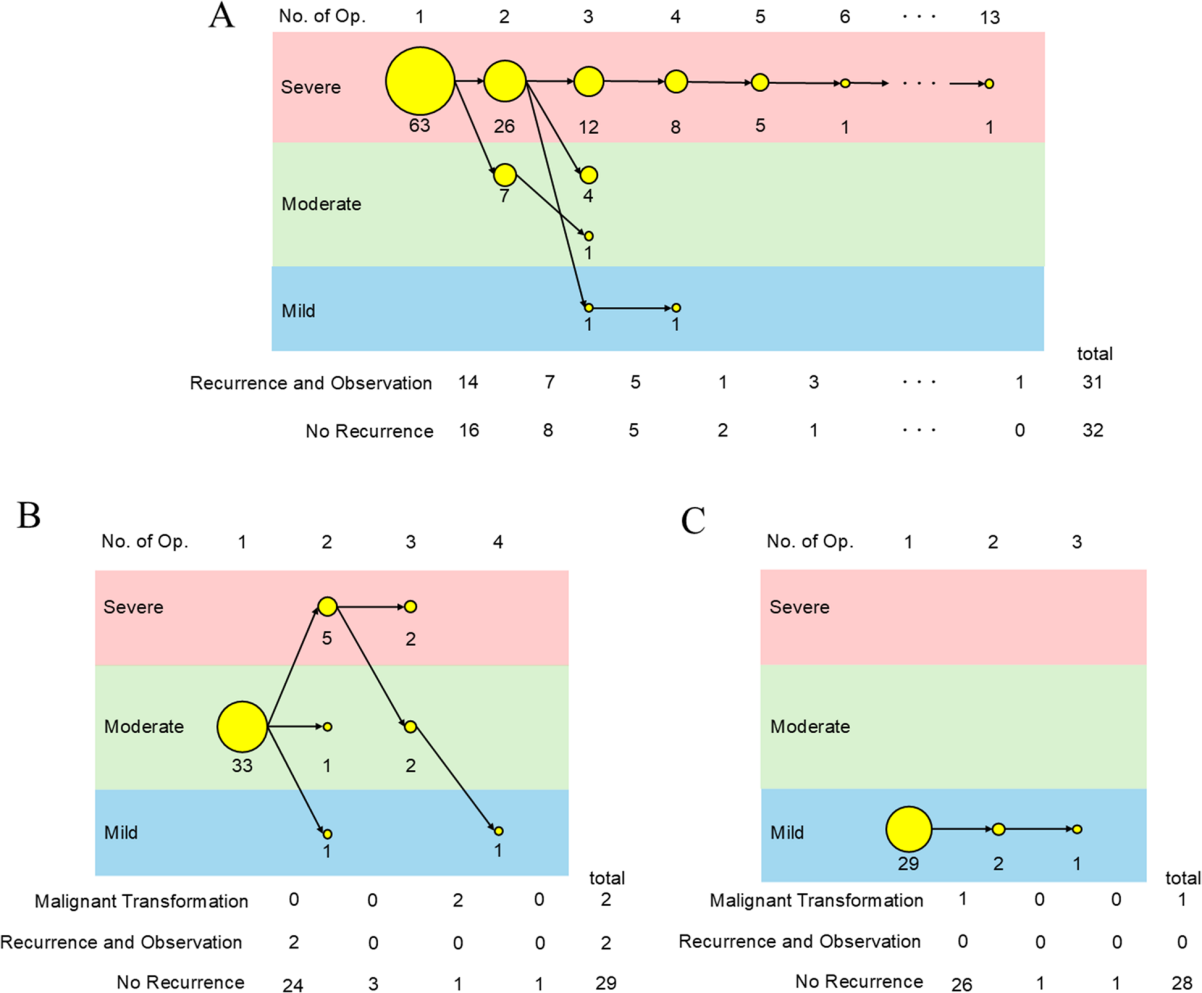
Clinical course over time. **A** Even in patients initially classified as Severe, disease severity tends to decrease over time, with reclassification to Moderate or Mild after repeated surgeries. Additionally, approximately half of the patients remain recurrence-free during follow-up. **B**: Patients classified as Moderate show a variable clinical course, with some progressing to Severe, some improving to Mild, and others undergoing malignant transformation. However, most patients with Moderate disease remain recurrence-free after a single surgical intervention. **C**: Patients classified as Mild generally do not experience recurrence. However, one patient demonstrates malignant transformation despite its initial classification

To further evaluate the clinical utility of the D-H Classification, we investigated the subsequent clinical course of patients stratified by disease severity at the time of initial surgery. Among the 63 patients initially classified as having Severe disease, the clinical outcomes during follow-up were as follows (Fig. 7A): 16 did not experience recurrence, 14 experienced recurrence but were managed with observation, and 33 required a second surgery. Of the 33 patients who underwent a second surgery, 26 remained in the Severe category, whereas seven were reclassified as Moderate. This classification process was repeated throughout the observation period. Ultimately, 32 patients did not exhibit recurrence, whereas 31 experienced recurrence but were under observation. Although one patient underwent as many as 13 surgeries, a general trend toward decreased severity with repeated interventions was observed.

In the Moderate group (Fig. 7B), 24 of 33 patients did not experience recurrence after the initial surgery. Similarly, in the Mild group (Fig. 7C), 26 of 29 patients remained recurrence-free after the first surgery. These findings suggest that both patients with Moderate and Mild disease have favorable prognoses and may be cured through surgical intervention alone. However, some patients with Moderate disease severity progressed to the Severe category during the follow-up, indicating the need for careful and continuous monitoring.

We also performed an analysis of the D–H classification restricted to HPV-positive cases (Additional file 8: Fig. S5A-C). In the severe, moderate, and mild groups, the trends observed were similar to those mentioned above, which included both HPV-positive and HPV-negative cases.

Notably, malignant transformation was observed in two patients in the Moderate group and one patient in the Mild group. It should be noted that two cases were HPV-negative and represent a pathophysiology distinct from RRP. Unexpectedly, no patients presented with malignant transformations in the Severe disease group. In clinical settings where routine HPV testing is not available, although infrequent, HPV-negative papillomas in cases classified as Moderate or Mild could potentially undergo malignant transformation over the course of the disease. Nevertheless, the use of the HARRP Score and D–H classification for all papillomatous lesions, including RRP, without HPV testing may still allow for more accurate assessment of prognosis than was previously possible.

## Discussion

RRP often presents significant challenges for both patients and healthcare providers. In patients where disease progression cannot be controlled by HPV vaccination or surgery, the administration of bevacizumab, a molecular-targeted agent supported by an international consensus statement [8], may be considered. This represents an attempt to use molecular-targeted therapy for benign tumors, making the assessment of disease severity a critical issue. The Derkay Score is considered the gold standard for evaluating the severity of RRP. However, severity scores vary depending on the criteria used as indicators. This study reports that a score as low as 4, which is substantially lower than the previously utilized cut-off value of 20 [17], may serve as an important predictor of recurrence. Although interrater variability in the Derkay Score does not pose a statistically significant issue [17], a notable limitation is the inherent difficulty in achieving complete agreement among evaluators. To address this issue, we proposed the HARRP Score, which is an objective evaluation method based on pathological findings. Moreover, by combining the HARRP Score with the Derkay Score, we developed the D-H Classification, which enables classification into three severity groups.

Most of the patients in this study were male with a history of smoking and alcohol consumption. This patient profile is consistent with that observed in previous studies and is therefore not unique to the present study [2, 33]. Surgical treatments commonly used include sharp excision (referred to as the “cold method”), CO₂ laser, and micro-debridation. The selection of method is often influenced by factors such as the advantages and disadvantages of each technique [34] and treatment decisions may affect the study outcomes. As this study was a multicenter collaborative research effort involving 16 institutions across Japan, rather than being limited to a single center, surgeries were performed by surgeons with varying levels of experience using different techniques, which likely reduced the potential bias related to the surgical methods. In addition, only patients requiring complete macroscopic tumor resection aimed at long-term remission (i.e., virtual cure) were included in the analysis. This facilitated the evaluation of recurrence risk, providing a unique aspect not reported in previous studies.

Histologically, papillomas present as papillary proliferations originating from the basal layer of the epithelium [35]. Typically, the tumor surface expresses HPV-L1, an HPV capsid protein. Observation of HPV-L1-positive areas using IHC in combination with SEM or TEM enabled the visualization of HPV particles. These findings were consistent with those of our previous study [22, 36]. Additionally, HPV-E4, a viral protein involved in capsid assembly, exhibits a distribution pattern concordant with that of HPV-L1. Notably, previous studies have reported that HPV-E4 exhibits relatively high mRNA expression levels among the structural HPV genes in papillomas [24]. Persistent HPV infection has long been recognized as a fundamental component of the pathogenesis of RRP. The present findings on HPV-L1 and HPV-E4 support the notion of sustained viral particle production and ongoing infections, suggesting a strong association with disease activity. This result is consistent with that of our previous study [22, 23]. However, the positivity rates for HPV-L1 and HPV-E4 on IHC were unexpectedly low at 20.0% and 39.4%, respectively. Given that patients who are positive for these markers tend to experience higher recurrence risk, these findings may indicate increased disease activity in such patients. To further assess disease activity, we evaluated the MIB-1 index based on the IHC staining of Ki-67. In cervical intraepithelial neoplasia (CIN), Ki-67 expression extends from the basal layer to the epithelial surface in correlation with an increasing histologic grade from CIN1 to CIN3 [37]. In the present study, we divided the epithelium into three layers (basal, intermediate, and superficial) and assessed the MIB-1 index. Our results demonstrated that a high MIB-1 index in the superficial third of the tumor epithelium was predictive of disease recurrence. We also investigated the utility of p16 IHC as a surrogate marker commonly used for HPV detection in oropharyngeal carcinoma [32]. Consistent with previous studies, p16 expression did not correlate with disease activity or RRP recurrence [22, 38]. Expression of the p16 protein typically requires the integration of HPV DNA into the host genome and overexpression of the HPV-E7 oncogene [39]. However, the low-risk HPV types most associated with RRP, namely HPV types 6 and 11, exhibited a low propensity for genomic integration [40]. In support of this, previous reports have demonstrated low mRNA expression levels of E6 and E7 oncogenes in papillomas [24]. These findings suggest that p16 is not a reliable biomarker of disease activity in patients with RRP.

Based on the IHC findings, we incorporated three markers, HPV-L1, HPV-E4, and the MIB-1 index in the superficial third of the tumor epithelium, into the HARRP Score, as they were considered to reflect the underlying pathophysiology of RRP and contribute to the prediction of recurrence. ROC curve analyses were performed in two ways: first, assuming real-world clinical settings where routine HPV testing is difficult, and second, focusing exclusively on RRP cases by restricting the analysis to HPV-positive patients. This approach allowed us to determine the cut-off values for each parameter. Notably, similar results were obtained whether the analysis was restricted to HPV-positive cases or included both HPV-positive and HPV-negative cases. Cases exceeding these thresholds were assigned 1 point per marker. Specifically, any detectable positivity for HPV-L1 or HPV-E4 was assigned 1 point each, while ≥ 5% positivity in the superficial third of the epithelium for the MIB-1 index was also assigned 1 point. Accordingly, the HARRP Score ranged from 0 to 3, with four possible stages of disease activity. The predictive performance of the HARRP Score for disease recurrence was evaluated using ROC analysis in both the test and validation cohorts. This approach confirms the predictive accuracy and reproducibility of the scores. The optimal cut-off value for recurrence risk was determined to be ≥ 1. In practical terms, this implies that the presence of any one of the following—HPV-L1 positivity, HPV-E4 positivity, or ≥ 5% positivity in the superficial third of the MIB-1 index—should be regarded as an indicator of elevated recurrence risk. We also performed ROC analysis for the Derkay Score, which yielded an optimal cut-off value of 4. In terms of the predictive accuracy of the Derkay Score, it was comparable, and in some cases slightly superior, to the HARRP Score. Multivariate analysis further revealed that both the HARRP and Derkay Scores were independently associated with recurrence. This may be the result of the small number of pediatric patients included in this study. Additionally, Kaplan–Meier survival analysis showed that both scoring systems produced statistically significant differences in recurrence-free intervals. Although many studies have indicated that a younger age is associated with disease severity [41, 42], age was not a contributing factor for recurrence in this study. These results indicate that the recurrence of RRP can be predicted using two complementary approaches: the Derkay Score, which reflects clinical severity [16], and the HARRP Score, which is based on pathological evaluation. The fact that similar results were observed both when restricting the analysis to HPV-positive cases and when including HPV-negative cases is particularly important.

Considering the recurrence-predictive capabilities of both the Derkay and HARRP Scores, we proposed a novel classification system, termed the D-H Classification, that integrates these two complementary approaches to further stratify disease severity in RRP. Categorizing patients into three groups—Severe (both positive scores), Moderate (either positive score), and Mild (both negative scores)—provides a framework for assessing disease activity. Kaplan–Meier analysis revealed that this stratification effectively distinguished the three groups without consideration of HPV status in terms of recurrence-free survival, underscoring its clinical utility. This integrative approach leverages the strengths of both scoring systems and reflects the anatomical and symptomatic disease burden, whereas the HARRP Score incorporates objective pathological and virological markers. Notably, the D-H Classification enables clinicians and researchers to identify patients at high risk of recurrence with greater precision, potentially informing follow-up intensity and treatment planning. Given the chronic and recurrent nature of RRP [41], a dual-parameter severity index, such as the D-H Classification, may serve as a valuable tool in routine clinical assessment and in the design of future clinical trials aimed at evaluating novel therapeutic interventions.

We further investigated the clinical course of patients stratified by the D-H Classification to gain insight into the natural history and treatment response in each subgroup. Among patients classified as Severe at the time of the initial surgery, many required multiple surgical interventions. However, approximately half of these patients eventually achieve disease remission as the tumor burden becomes increasingly controlled over time. Traditionally, treatment for RRP has prioritized the preservation of vocal fold function over complete tumor resection [43] based on the premise that a definitive cure is rarely achieved [41, 44]. In this study, surgical procedures were performed with careful consideration to preserve vocal fold function and avoid damage to the vocal ligament, and did not involve wide resection margins, akin to oncologic surgery. All the macroscopically visible lesions were excised. Although quality of life, particularly phonatory outcomes, was not directly assessed in this study, our findings suggest that gross total resection, when performed with attention paid to preserving the vocal fold architecture, may be a viable treatment option. Patients in the Moderate group at initial surgery demonstrated variable trajectories, with some transitioning to Severe or Mild, but nearly all ultimately achieving remission. In contrast, patients initially classified as having Mild disease exhibited a minimal risk of recurrence, which is consistent with the classification’s predictive intent. Contrary to our initial hypothesis, an unexpected and noteworthy observation was the occurrence of malignant transformation in patients classified as Moderate and Mild, rather than Severe. The cases that underwent malignant transformation during the course of disease were HPV-negative, suggesting a disease process distinct from typical HPV-positive RRP [30].　This finding highlights the potential limitations of severity scoring systems in predicting malignant progression, and suggests the presence of distinct underlying mechanisms. Further investigations are warranted to elucidate the mechanisms contributing to malignant transformation in these seemingly low-risk cases. Nonetheless, our findings serve as a cautionary note that even patients with Moderate or Mild disease may require careful long-term follow-up.

**Fig. 8:**
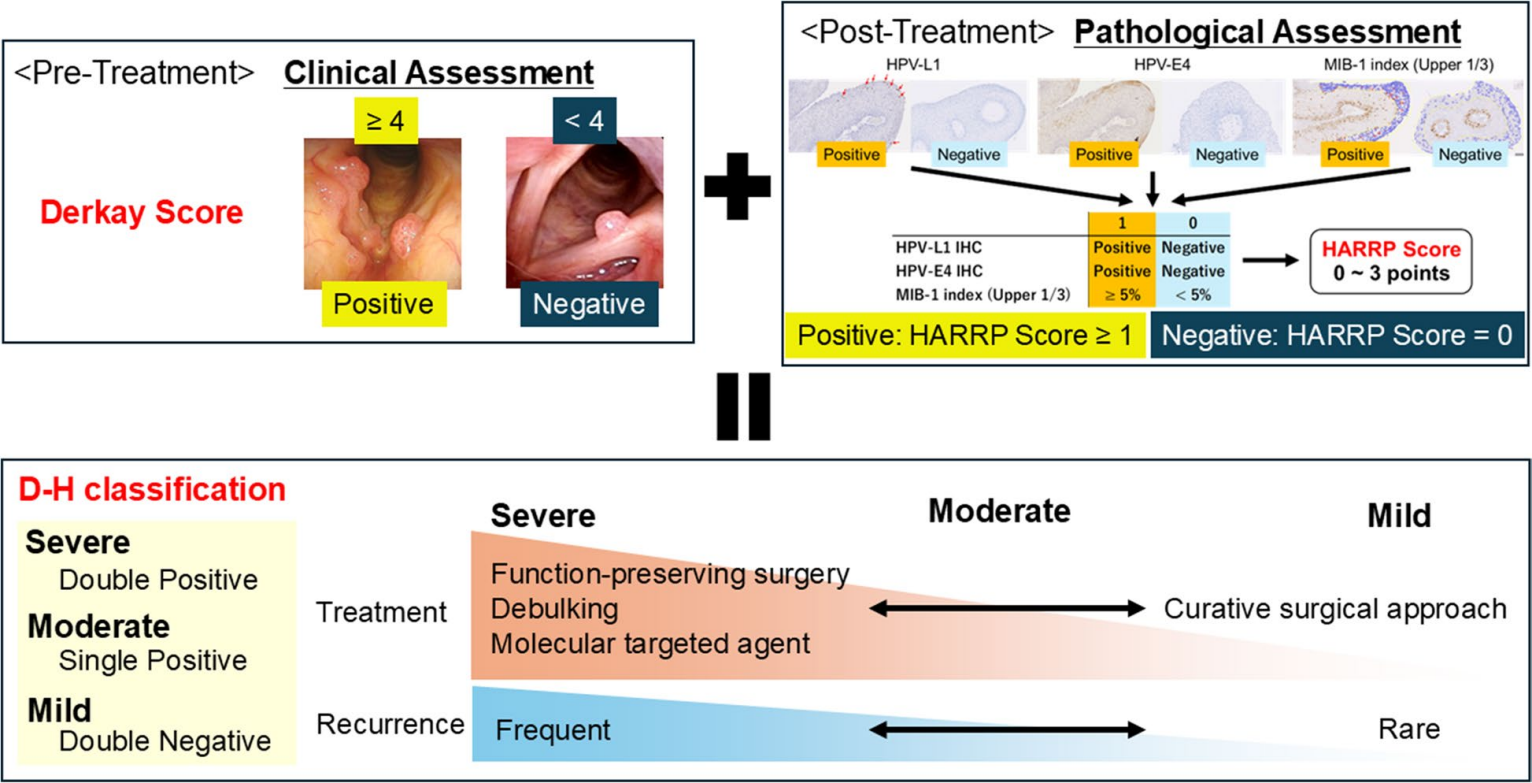
Workflow for severity assessment using D-H Classification. Prior to treatment, severity is assessed using the Derkay Score. A score of 4 or higher corresponds to Moderate or higher severity according to the D-H Classification, indicating the need for careful management. Post-treatment, the HARRP Score is adapted to evaluate disease status. Based on the D-H Classification after treatment, treatment strategies can be considered while taking the risk of recurrence into account. HARRP Score, Hamamatsu Recurrent Respiratory Papillomatosis Pathological Score; D-H Classification, Hamamatsu Recurrent Respiratory Papillomatosis Pathological Score combined with the Derkay Score Classification

The implementation of the HARRP Score and the D-H Classification offers a novel framework for severity assessment and follow-up strategies in RRP (Fig. 8). Before treatment, clinicians should rely on Derkay Scores. When the Derkay Score is ≥ 4, the final D-H Classification is likely to be Moderate or Severe, suggesting a clinically significant disease burden. Conversely, a Derkay Score < 4 implies that the disease is more likely to fall into a Mild or Moderate category. This information can assist in determining whether a curative (or long-term remission-oriented) surgical approach should be considered, or if a more conservative strategy focusing on airway security via debulking is appropriate. Following surgery, applying the HARRP Score based on IHC evaluation allows for a more precise classification. In patients with Severe disease, recurrence is anticipated, and clinicians can proactively plan further interventions, such as repeat surgery or adjuvant therapies such as bevacizumab. Although recurrence cannot be excluded in patients with Moderate disease, intensive surveillance may not be required. In patients with Mild disease, the risk of recurrence is minimal, potentially allowing for early discontinuation of follow-up. A major advantage of the HARRP Score is its simplicity and practicality. Assessment of HPV status is not required for using the HARRP Score and D-H classification, and the system can be applied to papillomatous lesions, including HPV-negative cases. HARRP Score relies solely on IHC staining, which is a widely available and routine pathological technique. Because the scoring criteria are straightforward, the burden on pathologists is minimal, making it an easily adaptable tool in most clinical settings.

Nonetheless, this study had some limitations. In clinical practice, not all surgical specimens from patients with RRP are submitted for comprehensive pathological evaluation. In patients where CO_2_ laser ablation or micro-debridation are used, the tissue may be insufficient or unavailable for analysis. Moreover, intralesional heterogeneity has been reported in RRP [45], and very small specimens may not allow for accurate scoring. Another consideration is the unique patient population of Japan. The low HPV vaccination rate in Japan may influence the disease characteristics [46, 47]. Notably, all patients in this study were unvaccinated; therefore, the utility of the D-H Classification in HPV-vaccinated individuals remains unknown. In addition, the HPV-E4 antibody used in this study is not currently commercially available. This antibody was generated by Taro Ikegami, a co-author of this manuscript [25]. At present, the antibody can be provided to researchers upon request.　Furthermore, evaluation of the superficial one-third of the MIB-1 index is not a common practice for pathologists and requires close collaboration. In practical use, what matters is whether the 5% cut-off is exceeded; for cases in which this can be visually apparent, precise quantification of the MIB-1 index is not necessary. An inherent limitation of this study was its retrospective design. Prospective studies are necessary to validate the utility of the D-H Classification. Despite these limitations, our findings suggest that the HARRP Score and its integration into the D-H Classification shows significant promise as a new, objective method for assessing disease severity in RRP.

## Conclusions

Therefore, we propose the HARRP Score as a novel pathology-based severity grading system for RRP. By combining the HARRP Score with the Derkay Score, the D-H Classification enables clinically meaningful stratification and may help guide both treatment selection and follow-up strategies. This dual-parameter approach may support tailored treatment decisions and individualized follow-up protocols, ultimately improving clinical outcomes in patients with RRP. Future research should focus on standardizing the minimum amount of tissue required for a reliable HARRP Score assessment and whether the D-H Classification can be applied clinically to HPV-vaccinated individuals.

## Supplementary Information

Additional file 1

Additional file 2

Additional file 3

Additional file 4

Additional file 5

Additional file 6

Additional file 7

Additional file 8

## Data Availability

The datasets generated and/or analyzed during the current study are not publicly available but are available from the corresponding author on reasonable request.

## Abbreviations

AUC: Area under the curve
CI: Confidence interval
CIN: Cervical intraepithelial neoplasia
CLEM: Correlative light and electron microscopy
DAB: 3,3'-Diaminobenzidine
D-H Classification: Derkay-HARRP Classification
FFPE: Formalin fixed paraffin embedded
HARRP Score: Hamamatsu Recurrent Respiratory Papillomatosis Score
HPV: Human papillomavirus
HR: Hazard ratio
IHC: Immunohistochemistry
IRB: Institutional review board
PCR: Polymerase chain reaction
ROC: Receiver operating characteristic
RRP: Recurrent respiratory papillomatosis
SD: Standard deviation
SEM: Scanning electron microscopy
YAG-BSE: Yttrium–aluminum-garnet backscattered electron
