# Supplementary material for: Development of a novel prognostic assessment tool for recurrent respiratory papillomatosis
Source: BMC Med. 2026 Apr 16;24:219. doi: 10.1186/s12916-026-04832-w (PMC13085680; doi:10.1186/s12916-026-04832-w)
Supplement: Supplementary file 1 — Additional file 1 [file 12916_2026_4832_MOESM1_ESM.docx]

**Table S1. Ethics approval of all facilities**

| Facility | Approval number |
| --- | --- |
| Hamamatsu University School of Medicine | 19–222 |
| Tottori University Faculty of Medicine | 23A068G |
| Kobe University School of Medicine | B230151 |
| Tokai University School of Medicine | 23R082-001 MH |
| Kanazawa University Graduate School of Medical Science | 114165-1 |
| Chiba University Graduate School of Medicine | HJK0244-23 |
| Niigata University Graduate School of Medical and Dental Sciences | 2022-0275 |
| University of the Ryukyus Graduate School of Medicine | 24-2411-00-00-00 |
| Shinshu University School of Medicine | 5837 |
| Mie University Graduate School of Medicine | H2023-073 |
| Kindai University Nara Hospital | 697 |
| Seirei Hamamatsu General Hospital | 3493 |
| Yaizu City Hospital | 302 |
| Numazu City Hospital | 2023-003 |
| Fujieda Municipal General Hospital | R-FY20-16 |
| Seirei Mikatahara General Hospital | 20-41 |
