# Supplementary material for: Development of a novel prognostic assessment tool for recurrent respiratory papillomatosis
Source: BMC Med. 2026 Apr 16;24:219. doi: 10.1186/s12916-026-04832-w (PMC13085680; doi:10.1186/s12916-026-04832-w)
Supplement: Supplementary file 2 — Additional file 2 [file 12916_2026_4832_MOESM2_ESM.docx]

**Table S2. Parameter Settings for the “Positive Cell Detection” Function in QuPath**

| Parameter | | Setting |
| --- | --- | --- |
| Setup parameters | |  |
|  | Detection image | Optical density sum |
|  | Requested pixel size | 0.5 μm |
| Nucleus parameters | |  |
|  | Background radius | 6 μm |
|  | Use opening by reconstruction | Select |
|  | Median filter radius | 0 μm |
|  | Sigma | 1.5 μm |
|  | Minimum area | 10 μm^2^ |
|  | Maximum area | 400 μm^2^ |
| Intensity parameters | |  |
|  | Threshold | 0.05 |
|  | Max background intensity | 3 |
|  | Split by shape | Select |
|  | Exclude DAB (membrane staining) | unselect |
| Cell parameters | |  |
|  | Cell expansion | 3 μm |
|  | Include cell nucleus | Select |
| General parameters | |  |
|  | Smooth boundaries | Select |
|  | Make measurements | Select |
| Intensity threshold parameters | |  |
|  | Score compartment | Nucleus: DAB OD mean |
|  | Threshold 1+ | 0.2 |
|  | Threshold 2+ | 0.4 |
|  | Threshold 3+ | 0.6 |
