# Supplementary figures and images for: Development of a novel prognostic assessment tool for recurrent respiratory papillomatosis
Source: BMC Med. 2026 Apr 16;24:219. doi: 10.1186/s12916-026-04832-w (PMC13085680; doi:10.1186/s12916-026-04832-w)

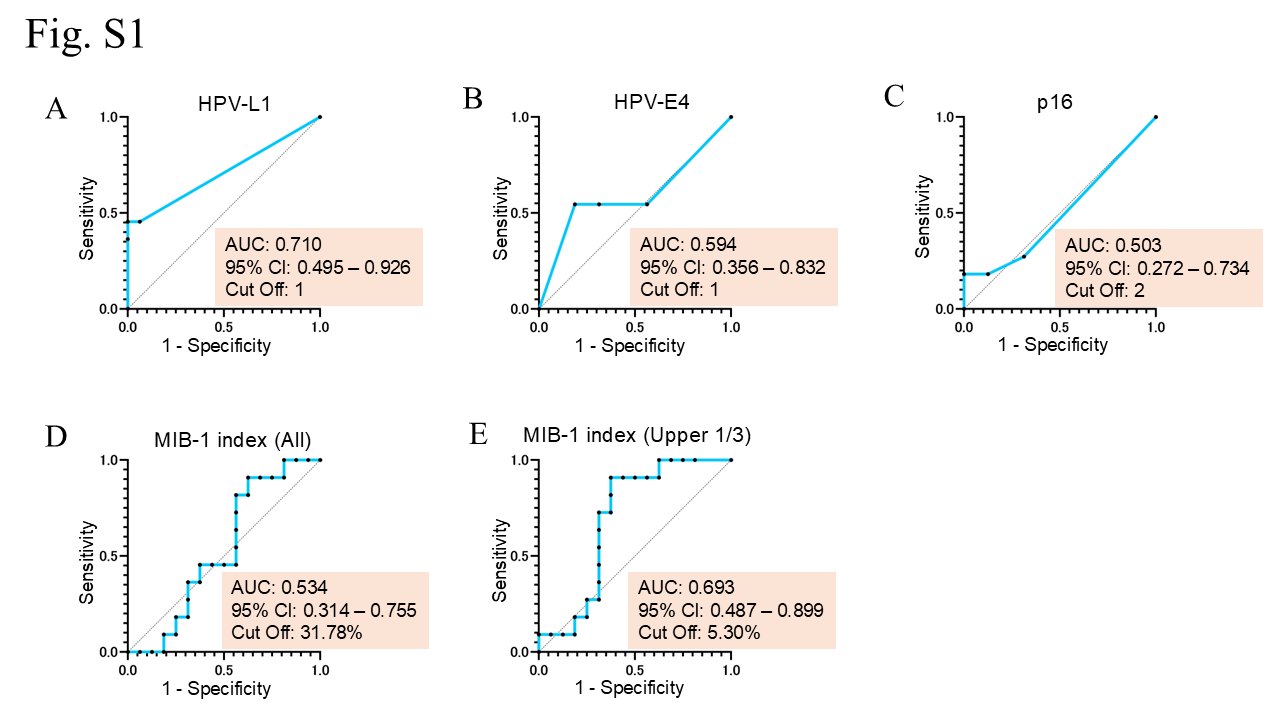

Supplement: Supplementary file 3 — Additional file 3 [file 12916_2026_4832_MOESM3_ESM.tif]

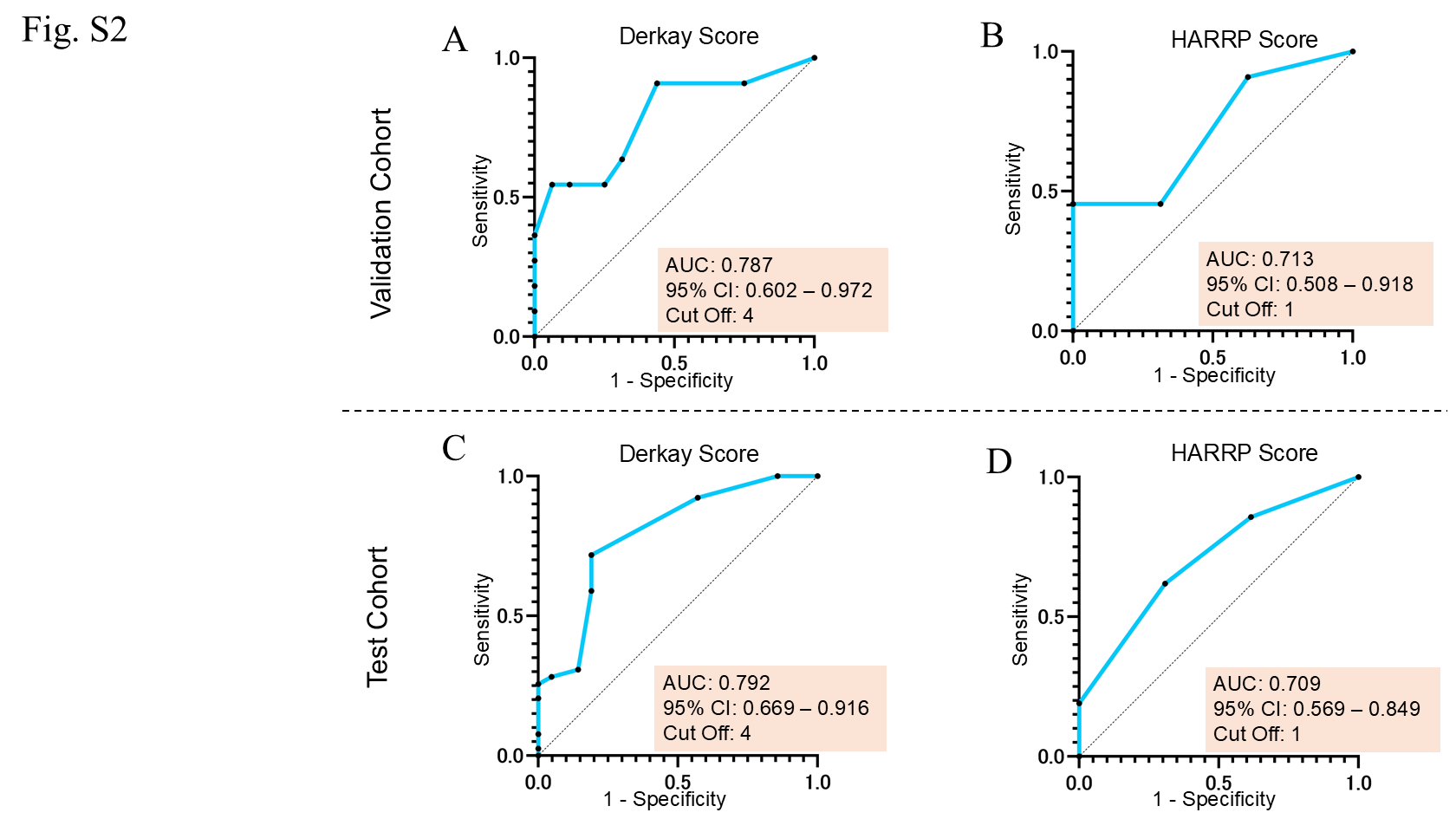

Supplement: Supplementary file 4 — Additional file 4 [file 12916_2026_4832_MOESM4_ESM.tif]

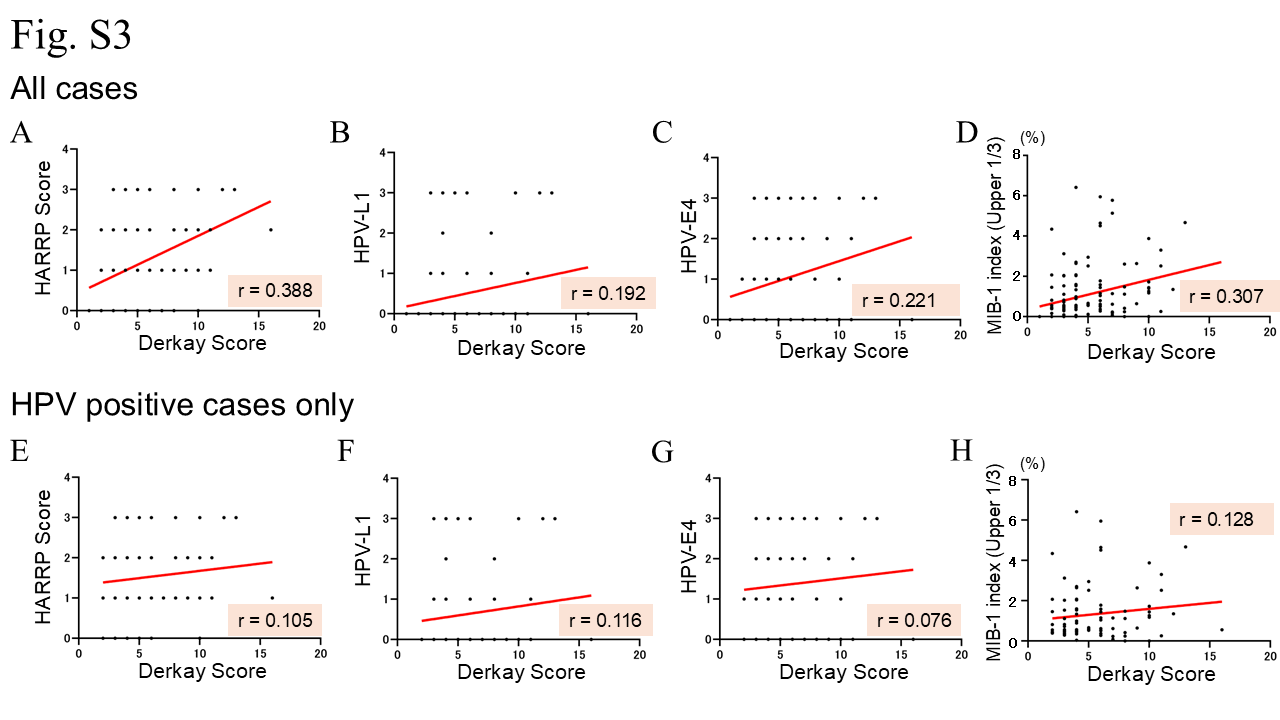

Supplement: Supplementary file 6 — Additional file 6 [file 12916_2026_4832_MOESM6_ESM.tif]

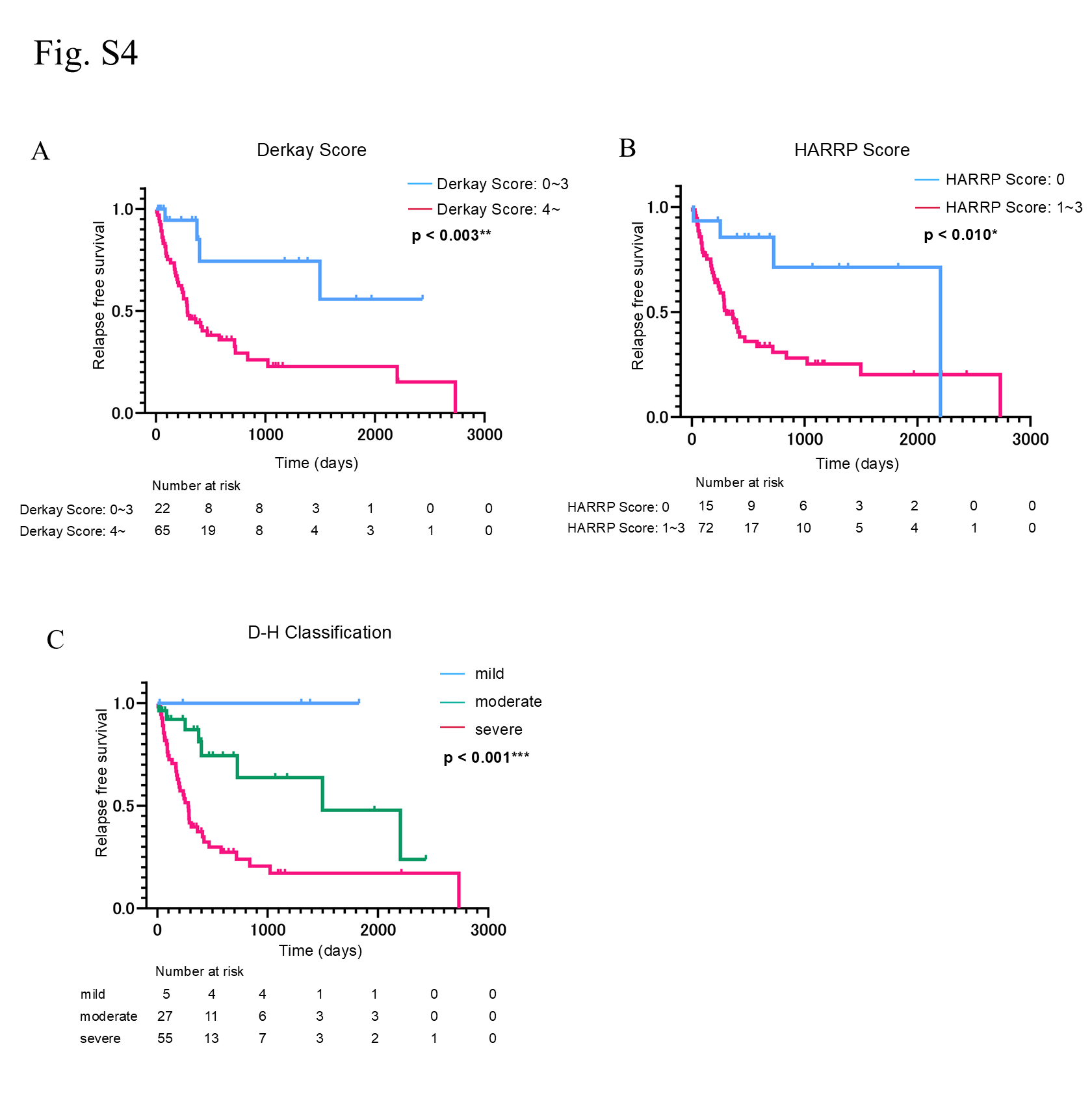

Supplement: Supplementary file 7 — Additional file 7 [file 12916_2026_4832_MOESM7_ESM.tif]

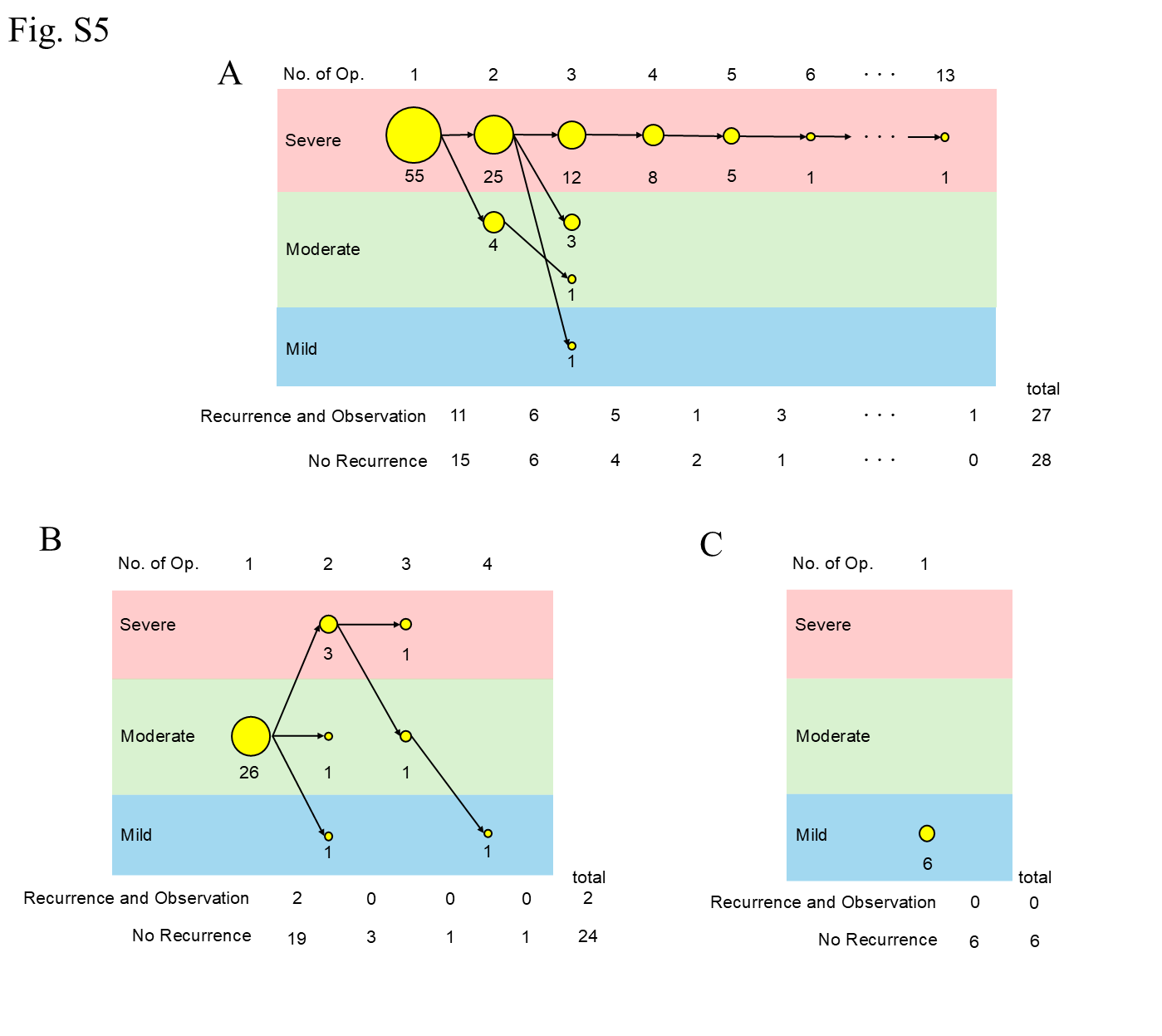

Supplement: Supplementary file 8 — Additional file 8 [file 12916_2026_4832_MOESM8_ESM.tif]
