# Supplementary material for: Development of a novel prognostic assessment tool for recurrent respiratory papillomatosis
Source: BMC Med. 2026 Apr 16;24:219. doi: 10.1186/s12916-026-04832-w (PMC13085680; doi:10.1186/s12916-026-04832-w)
Supplement: Supplementary file 5 — Additional file 5 [file 12916_2026_4832_MOESM5_ESM.docx]

**Table S3. Cox proportional hazards analysis of Derkay and HARRP Scores in relation to recurrence among HPV-positive cases**

| Covariate | Univariate analysis | | Multivariate stepwise analysis | |
| --- | --- | --- | --- | --- |
|  | HR (95% CI) | p | HR (95% CI) | p |
| Age | 1.167 (0.617 - 2.210) | 0.634 | - | - |
| Sex | 1.391 (0.590 - 3.277) | 0.451 | - | - |
| Alcohol | 1.246 (0.620 - 2.504) | 0.537 | - | - |
| Smoking | 0.753 (0.451 - 1.818) | 0.337 | - | - |
| Derkay Score | 4.294 (1.533 - 12.03) | < 0.006** | 4.109 (1.466 - 11.520) | < 0.007** |
| HARRP Score | 3.528 (1.262 - 9.864) | < 0.016* | 3.339 (1.194 - 9.339) | < 0.022* |

Cox proportional hazards models are applied, adjusting for age (≥60 years vs. <60 years), sex, alcohol consumption, smoking, Derkay Score (≥4 vs. <4), and HARRP Score (≥1 vs. <1). **: p<0.01. *: p<0.05. HARRP Score, Hamamatsu Recurrent Respiratory Papillomatosis Pathological Score; CI, confidence interval; HR, hazard ratio.
